# Defective Regulation of Membrane TNFα Expression in Dendritic Cells of Glioblastoma Patients Leads to the Impairment of Cytotoxic Activity against Autologous Tumor Cells

**DOI:** 10.3390/ijms21082898

**Published:** 2020-04-21

**Authors:** Tamara Tyrinova, Olga Leplina, Sergey Mishinov, Marina Tikhonova, Evgeniya Dolgova, Anastasiya Proskurina, Vyacheslav Stupack, Sergey Bogachev, Alexander Ostanin, Elena Chernykh

**Affiliations:** 1 Institute of Fundamental and Clinical Immunology, 630099 Novosibirsk, Russia; oleplina@mail.ru (O.L.); martix-59@mail.ru (M.T.); ostanin62@mail.ru (A.O.); ct_lab@mail.ru (E.C.); 2 Institute of Medicine and Psychology, Novosibirsk State University, 630090 Novosibirsk, Russia; 3 Novosibirsk Research Institute of Traumatology and Orthopedics them. Y.L.Tsivyana, 630091 Novosibirsk, Russia; smishinov@yandex.ru (S.M.); stupack@yandex.ru (V.S.); 4 Federal Research Center Institute of Cytology and Genetics of Siberian Branch of the Russian Academy of Sciences, 630099 Novosibirsk, Russia; dolgova.ev@mail.ru (E.D.); asproskurina@gmail.com (A.P.); labmolbiol@mail.ru (S.B.)

**Keywords:** dendritic cells, glioblastoma, TACE/ADAM-17, TNFα, granule-dependent cytotoxicity

## Abstract

Besides an antigen-presenting function and ability to induce antitumor immune responses, dendritic cells (DCs) possess a direct tumoricidal activity. We previously reported that monocyte-derived IFNα-induced DCs (IFN-DCs) of glioblastoma multiforme patients express low levels of membrane TNFα molecule (mTNFα) and have impaired TNFα/TNF-R1-mediated cytotoxicity against immortalized tumor cell line HEp-2. However, whether the observed defect could affect killer activity of glioma patient DCs against autologous tumor cells remained unclear. Here, we show that donor IFN-DCs possess cytotoxic activity against glioblastoma cell lines derived from a primary tumor culture. Granule-mediated and TNFα/TNF-R1-dependent pathways were established as the main mechanisms underlying cytotoxic activity of IFN-DCs. Glioblastoma patient IFN-DCs showed lower cytotoxicity against autologous glioblastoma cells sensitive to TNFα/TNFR1-mediated lysis, which was associated with low TNFα mRNA expression and high TACE/ADAM-17 enzyme activity. Recombinant IL-2 (rIL-2) and human double-stranded DNA (dsDNA) increased 1.5-fold cytotoxic activity of patient IFN-DCs against autologous glioblastoma cells. dsDNA, but not rIL-2, enhanced the expression of TNFα mRNA and decreased expression and activity of TACE/ADAM-17 enzyme. In addition, dsDNA and rIL-2 stimulated the expression of perforin and granzyme B (in the presence of dsDNA), suggesting the possibility of enhancing DC cytotoxicity against autologous glioblastoma cells via various mechanisms.

## 1. Introduction

Dendritic cells (DCs) play a key role in antitumor immune responses due to their ability to present tumor antigens to naive T cells, as well as to induce generation of cytotoxic CD8+ T lymphocytes [1,2]. In the past 20 years, in addition to antigen-presenting function and the ability to stimulate immune responses, DCs have been shown to exhibit cytotoxic activity in that they can directly cause tumor cell death via granule- and death receptor-mediated mechanisms [3,4]. The former mechanism is associated with the release of perforin and granzyme B cytotoxic mediators from lytic granules of DCs [5,6,7]. The latter pathway involves the expression of cytotoxic molecules belonging to the tumor necrosis factor family (TNFα, FasL, TRAIL, etc.) on the DC surface, which trigger target tumor cell death by binding to relevant receptors [3,5,8,9]. Nevertheless, killer function of DCs is poorly understood, thus raising many questions regarding its role in antitumor immune responses.

Since monocytes act as a natural pool of progenitor cells for DCs in pathology, a substantial proportion of DCs within tumor microenvironment is monocyte-derived. Conventional models for in vitro studies of such DCs are based on DC cultures generated from monocytes in the presence of GM-CSF and IL-4 (IL-4-DCs) [10]. At the same time, an important role in the generation of monocyte-derived DCs is played by type I interferons, especially IFNα, which is produced by most cells in response to inflammatory stimuli and functions as a danger signal [11]. IFNα is able to trigger rapid differentiation of circulating monocytes into DCs [12]. Moreover, IFNα itself and IFNα-activated transcription factors (STAT1, IRF7, ISGF3, etc.) regulate not only most genes involved in DC differentiation [12], but also genes encoding cytotoxic molecules (TRAIL, perforin and granzymes) [5]. DCs differentiated in the presence of IFNα (IFN-DCs) represent a unique DC population, which differ from IL-4-DCs in possessing a more pronounced antigen-presenting ability, higher migration activity, and a more stable phenotype [13,14,15,16]. In addition, IFN-DCs exhibit cytotoxic activity against various tumor cell lines, express a wide range of cytotoxic ligands (TNFα, TRAIL, FasL, perforin), and also secrete granzyme B, which is barely produced by IL-4-DCs [5,9].

During tumor growth, functional DC activity is often impaired, which reduces the efficiency of antitumor immune responses. Our previous studies showed that IFN-DCs from patients with high-grade gliomas (glioblastoma multiforme) in contrast to DCs derived from patients with low-grade gliomas are characterized by impaired TNFα/TNF-R-dependent cytotoxicity. This defect manifests itself in the reduced ability of IFN-DCs to lyse HEp-2 laryngeal carcinoma cells (which are selectively sensitive to TNFα/TNF-R1-mediated lysis), and this defect has been shown to be associated with low expression levels characteristic of the membrane form of TNFα (mTNFα) present on patient-derived DCs [17,18].

Taking into account that glioblastoma multiforme is characterized by an extremely unfavorable prognosis, even when intensive radiation and chemotherapy are used [19], one can assume that an impaired effector DC function could be one of the reasons for the aggressive tumor behavior. However, sensitivity of glioblastoma cells to DC-mediated cytotoxic activity remains poorly understood. In general, we envisage that the development of possible methods for regulating DC cytotoxic activity would be of great clinical interest. Indeed, the enhancement of DC-mediated cytotoxicity or obtaining DCs with high cytotoxic potential could constitute novel approaches in antitumor immunotherapy.

In the present study, we evaluate cytotoxic activity of IFNα-induced dendritic cells against glioblastoma cell lines obtained from primary tumor cell cultures, and investigated the mechanisms underlying DC-mediated glioblastoma cell lysis. The data obtained revealed a defect in TNFα/TNF-R1-dependent DC cytotoxicity against autologous tumor cells in glioblastoma patients. Therefore, we also elucidated molecular mechanisms of impaired cytotoxicity, as well as analyzed approaches to enhance effector DC functions.

## 2. Results

### 2.1. Glioblastoma Cell Lines Derived from Primary Tumor Cultures and Immortalized U87 Cell Line Differ in the Expression of TNF-Family Death Receptors

We used short-term cell lines obtained from primary glioblastoma cell cultures as a model for studying a cytotoxic potential of IFN-DCs. This approach allowed us to increase the number of tumor cells after several passages, as well as to obtain morphologically homogeneous cell populations. A standard immortalized U87 glioblastoma cell line served as a control culture in various series of experiments.

**Figure 1:**
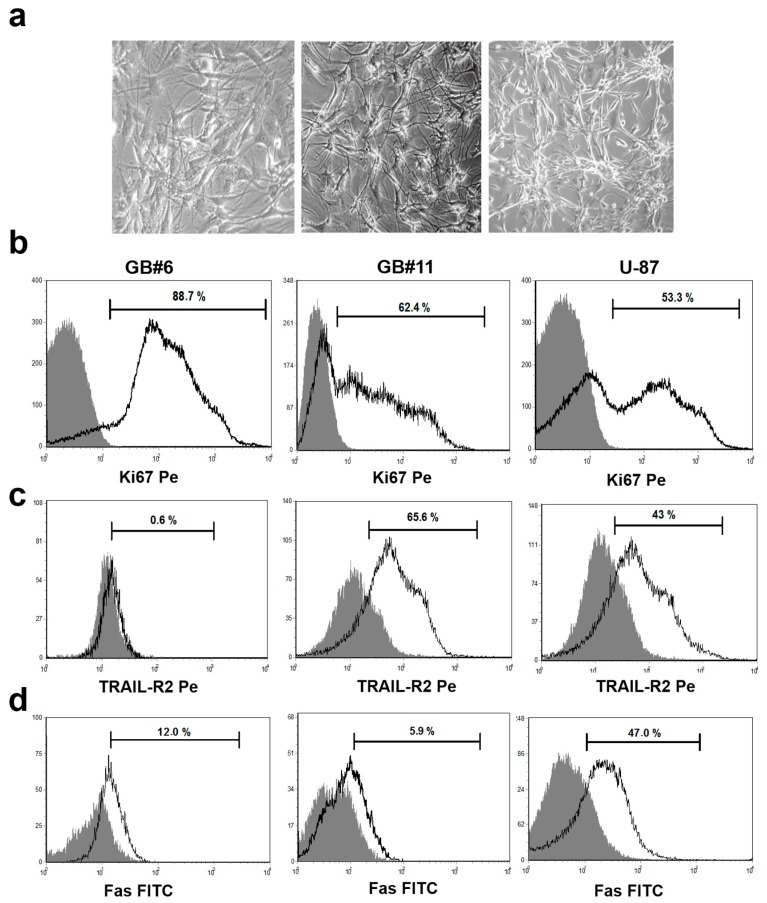
Glioblastoma cell lines derived from primary tumor cell cultures. (**a**) Figures show representative phase contrast images (×250 magnification) of cell lines derived from two primary glioblastoma cell cultures after 2–3 passages (left and middle panels) and human glioblastoma U-87 cells (right panel). (**b**–**d**) Flow cytometry histograms representing the expression of Ki-67 and TNF-family receptors (TRAIL-R2, Fas; bold-line histograms) and matched isotype controls (gray-filled histograms) in 2 of 13 cell lines derived from primary glioblastoma cell cultures (GB#6 and GB#11) after 2–3 passages (left and middle panels) and in human glioblastoma U-87 cells (right panel).

The particular glioblastoma cell lines studied here differed morphologically, but in general exhibited features characteristic of U87 cells (Figure 1a).

Glioblastoma cell lines had high proliferative activity, which remained at the same level during subsequent passages (Figure 1b). Proliferative activity (the relative number of Ki-67^+^ cells) ranged from 4.4% to 88.7% depending on a specific cell line (mean, 40.0% ± 21.2%). For some cell lines, this parameter even exceeded the level of U87 cell line proliferation (>43.0%).

We have previously shown that glioblastoma cells express both types of TNFα receptor: TNF-R1 and TNF-R2 [20]. Moreover, TNF-R2 receptor, which lacks death domain (DD) [21], was expressed on glioblastoma cells to a lesser extent than DD-containing TNF-R1 receptor.

As shown in Figure 1, glioblastoma cells also expressed other TNF-family receptors containing DD domain (TRAIL-R and Fas receptors). Glioblastoma cell lines were characterized by high expression levels of TRAIL-R2 (mean, 54.8% ± 18.8%; Figure 1c). Only one tumor cell line was found to be TRAIL-R2-negative. In all other cases, the ratio of TRAIL-R2^+^ cells in short-term glioblastoma cell lines studied varied from 22.0% to 95.0% depending on the cell line. U87 glioblastoma cells considered to be sensitive to TRAIL-mediated lysis [22] expressed TRAIL-R2 at a lower level than several non-immortalized glioblastoma cell lines.

Glioblastoma cells were also characterized by lower frequency of Fas receptor expression (mean frequency, 19.6 ± 8.1%; Figure 1d), than U87 cells (mean frequency, 36.0% ± 4.0%).

Thus, the presence of proapoptogenic receptors on primary glioblastoma culture-derived tumor cells indicates possible sensitivity of glioblastoma cells to receptor-mediated lysis. Furthermore, individual differences observed in the expression levels of the receptors studied here in glioblastoma cell lines obtained from different patients can determine different sensitivity levels of tumor cells to apoptosis.

### 2.2. Glioblastoma Cell Lines Are Sensitive to the Cytotoxic Effect of Donor IFN-DCs via an Apoptosis Induction Mechanism

**Figure 2:**
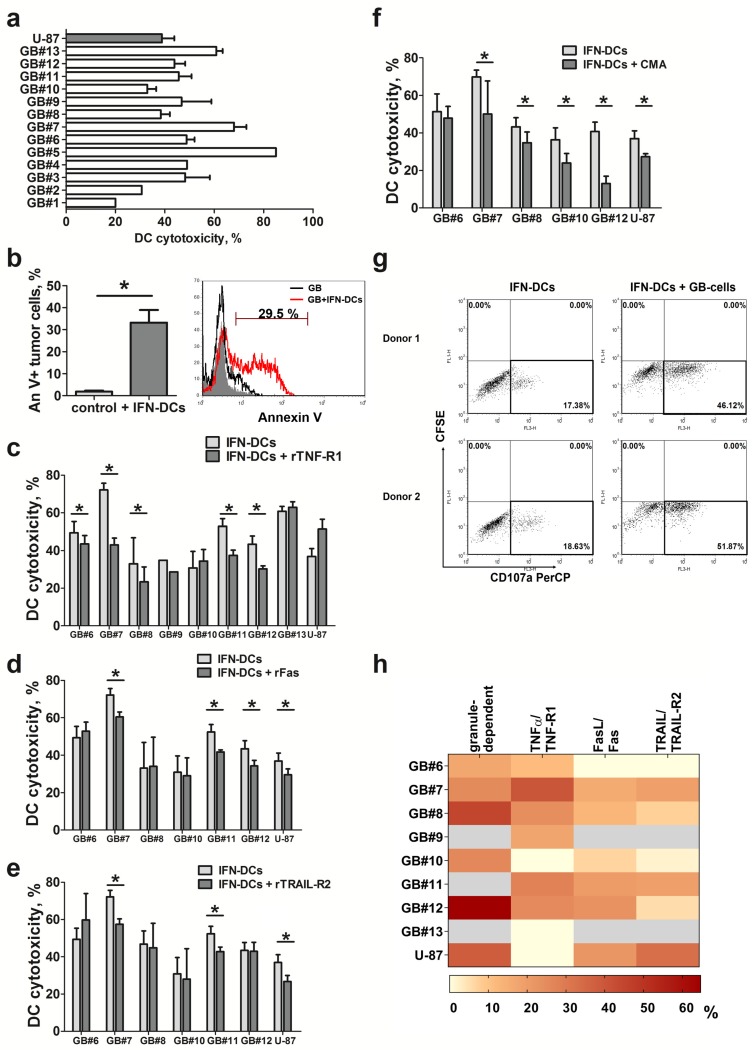
Cytotoxic activity of donor IFNα-induced DCs (IFN-DCs) against glioblastoma cell lines derived from primary tumor cell cultures. (**a**) Cytotoxic activity of healthy donor LPS-stimulated IFN-DCs against glioblastoma cell lines derived from primary tumor cell cultures (GB) is presented as mean (m ± SE; *n* = 3–15) or individual values (for GB#1, GB#2, GB#4, GB#5). (**b**) Percentages of Annexin V^+^ glioblastoma cells (GB#6 is shown as a representative cell line) shown in 5(6)-carboxyfluorescein diacetate N-succinimidyl ester (CFSE)^+^ gate in the absence (control) and presence of donor IFN-DCs (+ IFN-DCs) after co-culturing for 18 h at a 10:1 ratio (DCs: GB cells). Flow cytometry histogram shows data from one of five representative experiment. (**c**–**f**) The effect of soluble recombinant human TNF RI/TNFRSF1A Fc chimera protein (rTNFR1; c), recombinant ruman Fas/TNFRSF6/CD95 Fc chimera protein (rFas; (**d**)), recombinant human TRAIL R2/TNFRSF10B Fc chimera protein (rTRAIL-R2; (**e**)) and concanamycin A (CMA) (**d**) on cytotoxic activity of donor IFN-DCs against glioblastoma cell lines is shown as mean (m ± SE; *n* = 3–7) or individual values (for GB#9) in 24 h MTT-assay at a 1:1 ratio (effectors: target cells). (**g**) Flow cytometry dot plots represent two of four independent experiments; CD107a expression within CFSE-negative cell gate (IFN-DCs) is shown before and after co-culture with glioblastoma cell (GB#6) for 18 h at a 10:1 ratio (DCs: GB cells). (**h**) Heat map shows an inhibitory effect (median, percent) of rTNF-R1, rFas, rTRAIL-R2 and CMA on donor IFN-DC cytotoxicity. The inhibitory effect was calculated as [1-(DC_(LPS+inhibitor)_cytotoxicity/DC_LPS_ cytotoxicity)] × 100%, where “inhibitor” indicates rTNF-R1, rFas, rTRAIL-R2 or CMA. Gray fields indicate that no analysis was performed. * *p* < 0.05 significant differences.

As shown in Figure 2a, IFN-DCs derived from healthy donors lysed all 13 glioblastoma cell lines tested. Depending on the cell line, IFN-DC cytotoxicity values varied from 20% to 85%, which reflected individual differences in the sensitivity of glioblastoma cell lines to killing. In most cell lines (9 of 13), donor DCs had a pronounced ability to lyse glioblastoma cells (cytotoxicity > 40%). Moreover, cytotoxic activity of IFN-DCs against many glioblastoma cell lines was higher than that observed against immortalized U87 cells.

Unlike DCs, donor IFN-DC supernatants (at 25% concentration, *v*/*v*) possessed a weak cytotoxic activity (≤12%) against glioblastoma cell lines, and in addition IFN-DC supernatants did not lyse U87 cells (data not shown). Thus, direct contact between DCs and tumor cells was required to trigger IFN-DC cytotoxic activity against glioblastoma cells.

Co-cultivation of glioblastoma cells pre-labeled with vital dye CFSE and donor IFN-DCs (Figure 2b) lead to a marked increase in the relative number of Annexin V^+^ cells, mainly due to early apoptosis (Annexin V^+^ PI^−^). Thus, the cytotoxic effect of donor DCs was mediated via induction of apoptosis in glioblastoma cells.

### 2.3. Granule-Mediated and TNFα/TNF-R1-Dependent Lysis are the Predominant Mechanisms of IFN-DC Cytotoxicity against Glioblastoma Cell Lines Derived from Primary Tumor Cultures

Next, we addressed the role of individual death receptor-mediated mechanisms underlying the cytotoxic effect of IFN-DCs. To this end, we performed a series of experiments involving inhibition of TNFα, FasL and TRAIL ligands by pretreating donor IFN-DCs with soluble rTNF-R1, rFas and rTRAIL-R2 receptors (Figure 2c–e). The strongest inhibitory effect was observed when IFN-DCs were treated with the soluble rTNF-R1 receptor. Thus, inhibition of TNFα/TNF-R1-dependent pathway (Figure 2c) reduced donor DC cytotoxic activity in 6 of 8 cell lines tested (median suppression value, 33%). However, no decrease in DC cytotoxicity was observed after treating two glioblastoma cell lines (GB#10 and GB#13) and U87 cells with rTNF-R1, which indicated that these cell lines were resistant to TNFα-dependent lysis.

Inhibition of FasL/Fas-dependent pathway (Figure 2d) reduced donor DC cytotoxic activity against 3 of 6 cell lines tested (GB#7, GB#11 and GB#12). Median inhibitory effect of rFas on sensitive cells was 19.6%. The inhibitory effect of rFas on DC cytotoxicity was also observed in U87 cell cultures, which is consistent with the literature data on sensitivity of this cell line to FasL-dependent apoptosis [22].

Similar results were also obtained when inhibiting a TRAIL-dependent pathway. Reduced cytotoxic activity upon treatment of IFN-DCs with soluble rTRAIL-R2 receptor (Figure 2e) was also detected only in 3 of 6 glioblastoma cell lines tested (GB#7, GB#10 and GB#11) (median suppression, 21.0%) and in U87 cell line, which has been reported to belong to a group of TRAIL-sensitive tumor cells [22].

Since inhibition of death receptor-mediated signaling pathways either partially neutralized the cytotoxic effect of donor IFN-DCs or did not affect this function at all, we studied possible involvement of a perforin/granzyme B-mediated mechanism in the cytotoxic activity of IFN-DCs against glioblastoma cells. For this purpose, donor DCs were pretreated with concanamycin A (CMA), which is a specific inhibitor of vacuolar H^+^-ATPase. CMA is known to facilitate pH neutralization in lytic granules ultimately leading to perforin inactivation [23,24]. Treatment of DCs with concanamycin A (Figure 2f) reduced DCs cytotoxic activity against all cell lines tested (median inhibitory effect, 37%), as well as against U87 cells (median inhibitory effect, 27.3%).

We confirmed the involvement of a granule-mediated mechanism in IFN-DC cytotoxic activity against glioblastoma cell lines in DC degranulation experiments. Indeed, co-culturing donor IFN-DCs with glioblastoma cells (Figure 2g) resulted in more than a 3-fold increase in the number of DCs expressing CD107a (CD107a^+^CFSE^+^ cells), which represents a lytic granule membrane marker.

Thus, granule-mediated and TNFα/TNF-R1-dependent pathways are the main mechanisms underlying IFN-DC cytotoxic activity against most short-term glioblastoma cell lines obtained (Figure 2h), while FasL/Fas- and TRAIL/TRAIL-R2-dependent pathways are not critical, thus making a significantly smaller contribution to donor IFN-DC-mediated cytotoxic activity against glioblastoma cells.

Importantly, no correlation was observed between the level of the inhibitory effect mediated by neutralizing molecules studied and the expression of the corresponding receptors on tumor cells. Thus, differences in the sensitivity of tumor cells to death receptor-mediated lysis are not entirely related to the expression characteristics of these receptors. Moreover, the data obtained indicates that despite a similar histogenetic origin tumor cells from glioblastoma patients differ from immortalized U87 glioblastoma cells in their sensitivity to DC-mediated lysis and as far as particular pathways involved in this process.

### 2.4. Reduced Cytotoxic Activity of IFN-DCs from Glioblastoma Patients against Autologous and Allogeneic Glioblastoma Cells Sensitive to TNFα/TNF-R1-Mediated Lysis

**Figure 3:**
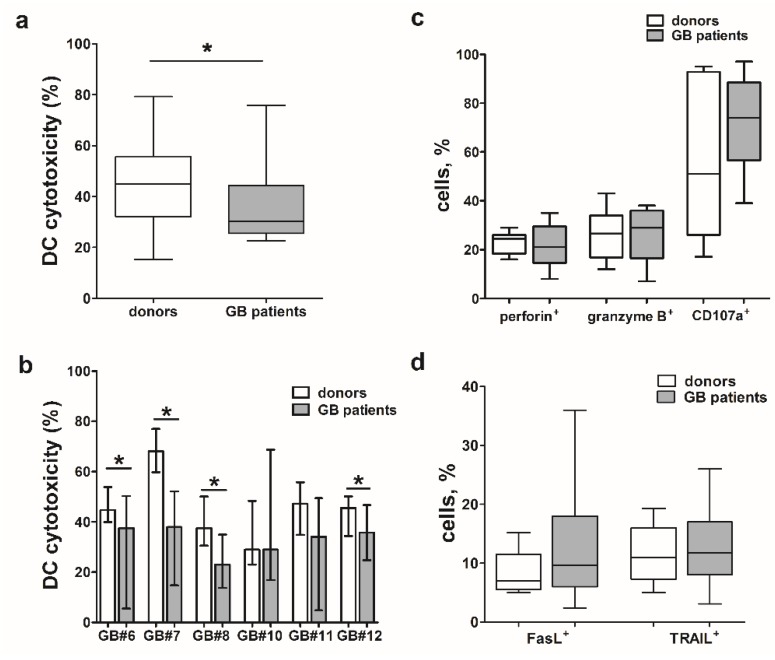
Cytotoxic activity of glioblastoma patient-derived IFN-DCs. (**a**) Data of cytotoxic activity of glioblastoma patient IFN-DCs against autologous glioblastoma cell lines derived from primary tumor cultures (*n* = 8) is presented as box and whisker plots (minimum to maximum values; median with interquartile range). Cytotoxic activity of donor IFN-DCs (*n* = 25) was analyzed against the same cell lines. (**b**) Cytotoxic activity of donor and patient IFN-DCs against allogeneic glioblastoma cell lines derived from primary tumor cultures is shown as mean (m ± SE; *n* = 2–4 individual experiments for each cell lines). (**c**,**d**) Data on intracellular (**c**) and surface (**d**) molecule expression (% of cells stained positively) obtained by flow cytometry in IFN-DCs of donors (*n* = 7–15) and patients (*n* = 7–15) is presented as box and whisker plots (minimum to maximum values; median with interquartile range). * *p* < 0.05 significant differences.

IFN-DCs generated from monocytes of glioblastoma patients were characterized by phenotypic profiles with delay of cell differentiation but expressed equal level molecules involved in antigen presentation (HLA-DR, CD86) to donor IFN-DCs as shown in Table 1 which confirmed our previous findings [25]. Compared to donor IFN-DCs, IFN-DCs from glioblastoma patients showed 30% lower cytotoxic activity towards the same glioblastoma cell lines (*p* = 0.05; Figure 3a).

In these experiments we tested DCs taken from 4–15 individuals against each cell line. Defective cytotoxic activity of IFN-DCs derived from glioblastoma patients against autologous tumor cells was observed only in the cell lines sensitive to TNFα/TNF-R1-mediated lysis (Table 2). However, if cells were not sensitive to TNFα/TNF-R1-dependent signaling pathway (GB#10, GB#13), cytotoxic activity of IFN-DCs derived from patients was comparable with that of donor origin.

Similar results were obtained for the cytotoxic activity of allogeneic IFN-DCs from glioblastoma patients against tumor cell lines studied (Figure 3b). Only when glioblastoma cells were resistant to TNFα-mediated lysis (GB #10), cytotoxic activities were the same for healthy donor-derived and patient-derived DCs. In all other cases, cytotoxicity of patient-derived DCs against allogeneic glioblastoma cell lines was lower, as compared that against the same tumor cells.

We found no correlations between cell line sensitivity to other TNFα/TNF-R1-independent mechanisms and defective cytotoxicity of patient DCs.

Thus, data obtained suggests that TNFα/TNF-R1-dependent mechanism plays an important role in DC-mediated cytotoxicity against autologous tumor cells and that defective TNFα-mediated DC cytotoxicity is responsible for reduced effector functions of patient IFN-DCs.

### 2.5. IFN-DCs from Glioblastoma Patients Do Not Differ from Donor IFN-DCs in the Molecular Expression Pattern of Intracellular Cytolytic Granules and Surface Ligands FasL and TRAIL

We have previously shown that IFN-DCs from glioblastoma patients are characterized by decreased expression of mTNFα, compared to donor IFN-DCs [18]. Here, we show that expression of other molecules involved in DC cytotoxic activity in glioblastoma patients is preserved. Thus, IFN-DCs from glioblastoma patients were comparable with donor IFN-DCs in terms of certain intracellular molecule expression in cytolytic granules, such as perforin, granzyme B, CD107a (Figure 3d), as well as surface molecules FasL and TRAIL (Figure 3c).

Hence, reduced mTNFα expression on IFN-DCs from glioblastoma patients could be a key factor responsible for the impaired cytotoxic DC function against tumor cells sensitive to TNFα/TNF-R1-dependent lysis. At the same time, the preserved expression of other ligands, mainly perforin and granzyme B, allows DCs from glioblastoma patients to lyse tumor cells, although at a lower level compared to the donor IFN-DCs.

### 2.6. Decreased Cytotoxicity of IFN-DCs from Glioblastoma Patients Is Associated with Low TNFα mRNA Levels and High TACE/ADAM-17 Activity

**Figure 4:**
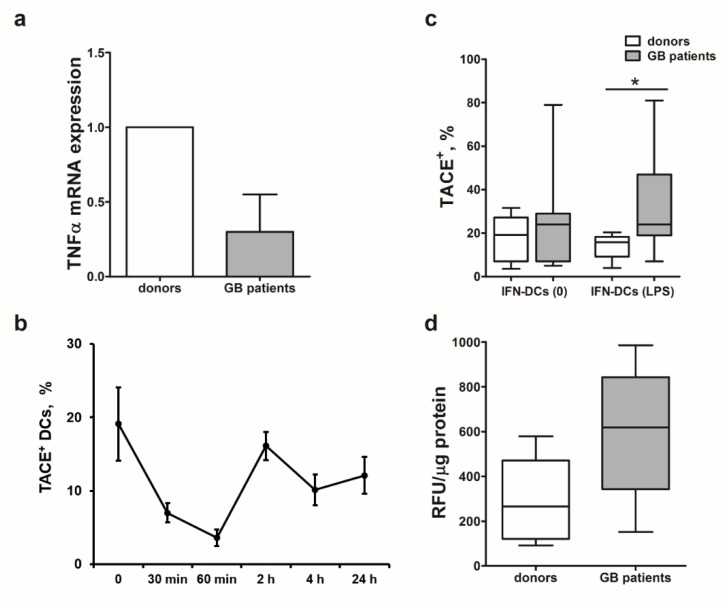
Expression of TNFα mRNA and a TNFα-converting enzyme in glioblastoma patient-derived IFN-DCs. (**a**) Relative levels of TNFα mRNA expression are presented as median values and interquartile range (LQ–UQ) normalized by a 2^−ΔΔ*C*t^ method using *RPLP0* reference gene in patient-derived IFN-DCs (*n* = 8); (**b**) TNFα-converting enzyme (TACE/ADAM-17) expression levels in unstimulated donor IFN-DCs and in response to LPS treatment are shown as mean values (m ± SE). Data were obtained from five independent experiments. (**c**) TACE/ADAM-17 expression on non-stimulated (0) and LPS-stimulated IFN-DCs derived from donors (*n* = 9) and patients (*n* = 8) are shown. (**d**) TACE/ADAM-17 enzyme activity in LPS-stimulated IFN-DCs from donors (*n* = 9) and patients (*n* = 8) are shown * *p* < 0.05 significant differences.

Next, we studied possible causes of mTNFα expression impairment ultimately leading to a selective defect in TNFα-mediated cytotoxic function of IFN-DCs from patients with high-grade gliomas against autologous tumor cells. IFN-DCs from glioblastoma patients were characterized by more than a twofold decrease in TNFα mRNA levels in comparison with donor IFN-DCs (Me 2^−ΔΔ*C*t^ = 0.35; *p* = 0.05; Figure 4a).

mTNFα expression also depends on the intensity of shedding and activity of a TNFα-converting enzyme (TACE/ADAM-17), which cleaves the extracellular part of mTNFα and converts it into biologically active secreted form of TNFα (sTNFα) [26]. Prior to comparing the expression of TACE/ADAM-17 in DCs from healthy donors and patients, we determined optimal checkpoints of enzyme detection in response to LPS stimulation (Figure 4b). An abrupt decrease in TACE/ADAM-17 levels was observed within the first 60 min after LPS was added to donor IFN-DC cultures. Subsequently, TACE/ADAM-17 expression levels increased reaching its maximum 2 h after stimulation initiation and then remained detectable up to 24 h of cultivation, followed by a gradual decrease. Based on the data obtained, we performed comparative analysis of TACE/ADAM-17 expression on IFN-DCs from healthy donors and glioblastoma patients 2 h after LPS treatment. Figure 4c shows that intact (LPS-unstimulated) DCs from patients were characterized by more pronounced expression of TACE/ADAM-17 enzyme, than donor DCs (*p* = 0.17). Moreover, TACE/ADAM-17 expression levels in LPS-stimulated DC cultures originated from patients were more than twofold higher, than healthy donor-derived DC (*p* = 0.035).

Maximum TACE/ADAM-17 activity in donor IFN-DCs observed in response to LPS stimulation corresponded to the highest expression level of this enzyme detected 2-h after treatment (data not shown). Similarly to increased TACE/ADAM-17 expression, patient-derived DCs were also characterized by a higher activity of TACE/ADAM-17 before (*p* = 0.2) and after (*p* = 0.08) stimulation with LPS, than donor DCs (Figure 4d). LPS-stimulated IFN-DCs from glioblastoma patients were characterized by nearly a threefold greater activity of TACE/ADAM-17, than donor-derived IFN-DCs.

The results obtained indicate that low mTNFα expression levels observed on DCs derived from glioblastoma patients could be attributed not only to the impaired TNFα expression, but also could be caused by high expression and activity levels of TACE/ADAM-17 responsible for shedding mTNFα from the DC membrane.

### 2.7. rIL-2 and dsDNA Up-Regulate IFN-DC Cytotoxic Activity against Autologous Glioblastoma Cells via Various Mechanisms

We have previously shown that recombinant interleukin 2 (rIL-2) and human double-stranded DNA (dsDNA) enhanced the expression of mTNFα on IFN-DCs from glioblastoma patients [18]. Our observations could be important for developing possible correction methods of impaired cytotoxic DC activity derived from glioblastoma patients against autologous tumor cells sensitive to TNFα/TNF-R1-dependent lysis.

**Figure 5:**
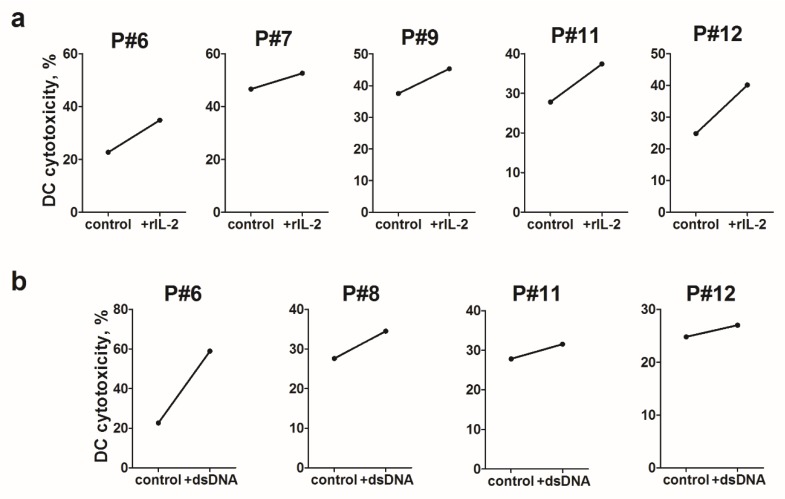
The effect of recombinant interleukin 2 (rIL-2) and human double-stranded DNA (dsDNA) on the cytotoxic activity of glioblastoma patient-derived IFN-DCs. Data are presented as individual values of patient-derived IFN-DC cytotoxic activity after stimulation with rIL-2 (**a**) or dsDNA (**b**) against autologous glioblastoma cell lines using MTT-assay at a ratio of effectors (DCs): targets (tumor cells) of 1:1.

Therefore, here we investigated the effects of rIL-2 and dsDNA as stimulators of cytotoxic activity of patient-derived DCs against glioblastoma cells. The stimulatory effect of rIL-2 and dsDNA on mTNFα expression was associated with an average of 1.5-fold increase in the cytotoxic activity of patient-derived IFN-DCs against autologous tumor cells (Figure 5).

**Figure 6:**
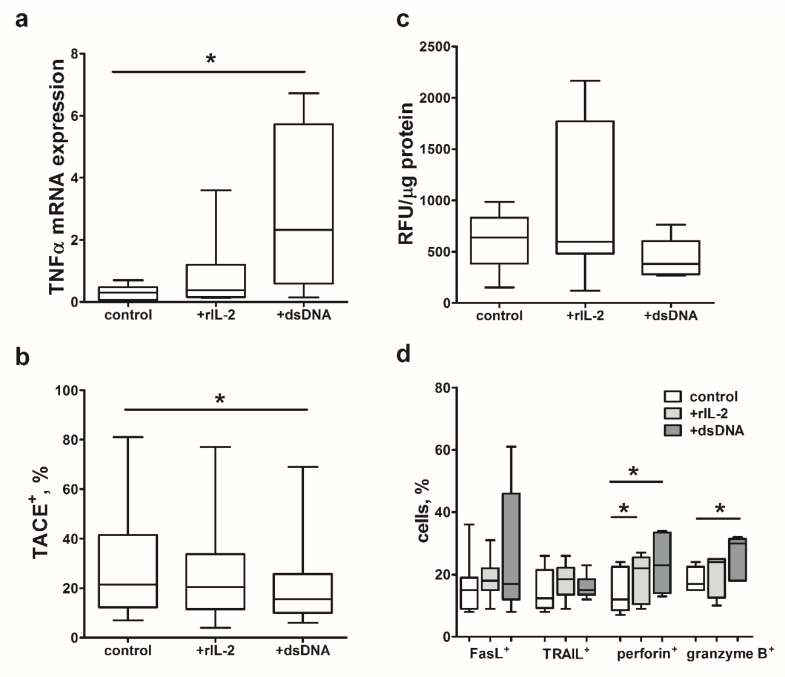
Effect of rIL-2 and dsDNA on glioblastoma patient-derived IFN-DCs. Data are presented as box and whisker plots (minimum to maximum values; median with interquartile range) of (**a**) TNFα mRNA expression, *n* = 9, (**b**,**c**) TACE/ADAM-17 expression (b, *n* = 7) and activity (c, *n* = 8), (**d**) surface (FasL, TRAIL, *n* = 8) and intracellular (perforin, granzyme B, *n* = 7) molecule expression (percentage of positively stained cells) in cultures of LPS-stimulated IFN-DCs (control) and stimulated with rIL-2 (+ rIL-2) or dsDNA (+ dsDNA) glioblastoma patient-derived IFN-DCs. * *p* < 0.05 significant differences.

Next, we studied the effect of rIL-2 and dsDNA at two potential levels of impaired regulation (transcriptional or post-translation) of mTNFα expression in patient-derived DCs (Figure 6a–c). Intact and rIL-2-treated IFN-DCs were characterized by an equally low level of TNFα mRNA expression (Figure 6a). In contrast to rIL-2, treatment of IFN-DC cultures with dsDNA increased TNFα mRNA expression in DCs (*p* = 0.05). In addition, dsDNA reduced TACE/ADAM-17 expression in patient-derived IFN-DCs and decreased its proteolytic activity by more than 20% (Figure 6b,c). We observed that rIL-2 did not affect TACE/ADAM-17 expression in IFN-DCs, altogether suggesting that rIL-2 and dsDNA regulate mTNFα expression through different mechanisms.

We also assessed a potential effect of rIL-2 and dsDNA on expression of other cytotoxic ligands involved in the cytotoxic DC activity against glioblastoma cells. Activation of IFN-DCs derived from glioblastoma patients with rIL-2 and dsDNA increased the expression of TNF-family mediators of receptor-dependent cytotoxicity (FasL and TRAIL), as well as granule-dependent cytotoxicity (Figure 6d). Interestingly, treatment with rIL-2 caused statistically significant increase in perforin expression and dsDNA—in both perforin and granzyme B expression.

## 3. Discussion

In this study, we have shown for the first time that IFNα-induced monocyte-derived DCs can act as effector cells and lyse glioblastoma cells obtained from primary tumor cultures. There is evidence to suggest that glioblastoma cells have low sensitivity to NK-mediated lysis. Indeed, glioblastoma cells express ligands of inhibitory NK cell receptors on their surface, while ligands of activating NK cell receptors are expressed at low levels [27,28,29]. In this study, cell lines obtained from primary glioblastoma cultures displayed various sensitivity to the cytotoxic effect of IFN-DCs, which generally reflects individual heterogeneity of glioblastoma. However, in most cases donor IFN-DCs showed an appreciably high level of cytotoxicity (>40%) against glioblastoma cells.

According to our observations on the expression patterns of death domain-containing TNF receptors on glioblastoma cell lines, the cytotoxic effect of IFN-DCs towards glioblastoma cell lines obtained from primary cultures can be mediated via external receptor-dependent apoptosis mechanisms. Data on the expression of TNF-R, TRAIL-R1/2 and Fas receptors on permanent human glioblastoma cell lines (SF-126, SF-188, U-138MG, LN235, etc.) and freshly isolated glioblastoma cells was reported previously [30,31,32]. However, our findings differ from the results of these studies, which demonstrated direct correlation between the sensitivity of glioblastoma cells to receptor-mediated mechanisms of apoptosis and the expression of the corresponding receptors [32,33]. In contrast to these observations, we found no correlation between the expression of TNFα, FasL and TRAIL receptors on tumor cells and the inhibitory effect of soluble receptors for these ligands. Hence, TNF-R, Fas and TRAIL-R receptors can not only be involved in apoptosis induction, but also in mediation of other effects, even serving as growth factors for glioblastoma cells [34].

In addition, we analyzed mediators of DC cytotoxic activity expressed on the cell surface of IFN-DCs. Thus, inhibition of cytotoxic ligands/mediators revealed that two main pathways underlie cytotoxic activity of donor IFN-DCs against most glioblastoma cells, i.e., granule-dependent and TNFα/TNF-R1-dependent pathways.

Granule-mediated cytotoxicity can play an important role in antitumor immunity in brain glioma tumors, owing to the fact that glioma cell lines can be resistant to death receptor-mediated apoptosis [35,36].

An important observation was made in the present study in that among the receptor-mediated pathways studied it was TNFα/TNF-R1-dependent mechanism that was involved in the vast majority of cases in lysis of glioblastoma cells by dendritic cells. Hence, this mechanism makes a significant contribution to the cytotoxic potential of IFN-DCs. IFN-DCs from glioblastoma patients were characterized by reduced cytotoxic activity against autologous tumor cells. Interestingly, the level of reduction in most cases corresponded to the intensity of rTNF-R1-mediated inhibitory effect, i.e., accounted for a fraction of the total cytotoxic potential mediated by TNFα/TNF-R1-dependent mechanism. We envisage that low mTNFα expression levels on IFN-DCs derived from glioblastoma patients reported in our previous study [18] could be a putative key factor contributing to the impairment of the cytotoxic DC activity against autologous tumor cells.

However, we observed the absence of total cytotoxic activity suppression against autologous tumor cells due to the impaired mTNFα expression, as evidenced by the expression of other mediators (perforin, granzyme B, FasL and TRAIL) in glioblastoma patient-derived IFN-DCs cultures was preserved at the levels comparable with that of donor IFN-DCs. Meanwhile, we cannot rule out the involvement of other receptor- and granule-independent mechanisms of DC cytotoxicity.

An important conclusion of this study could be drawn with regard to the immortalized U87 cell line, which differs from most non-immortalized glioblastoma cell lines in terms of its sensitivity to IFN-DC-mediated cytotoxic effect and lysis mechanisms involved. In particular, this cell line is resistant to TNFα/TNF-R1-mediated DC cytotoxicity. We have previously shown that IFN-DCs derived from patients with high-grade gliomas are comparable to healthy donor-derived IFN-DCs in terms of cytotoxic activity against U87 cells [17]. These results suggest that U87 cells are not an adequate analog of glioblastoma cells as an in vitro model and hence cannot be used as an optimal model for studying immune mechanisms of antitumor defense, as well as for developing novel antitumor drugs.

According to our data, reduced cytotoxic activity of glioblastoma patient-derived DCs was associated with low TNFα mRNA levels. According to S. Hira et al., DCs derived from patients with myeloid leukemia were also characterized by low cytotoxic activity against TNFα-sensitive tumor targets due to low *TNFα* gene expression in DCs [4]. Therefore, our results provide additional confirmation of the impaired TNFα-mediated cytotoxic activity of DCs in patients with malignant tumors.

In this study, we analyzed mTNFα expression regulation both at the transcriptional and post-translational levels to demonstrate for the first-time high expression and activity of TACE/ADAM-17 in IFN-DCs from glioblastoma patients. Similar changes in TACE/ADAM-17 activity were also found in high-grade glioma cells being associated with tumor progression and poor prognosis [37]. Our data indicate that TACE/ADAM-17 activity changes in patients with high-grade gliomas not only in tumor cells, but also in immune cells. This systemic impairment of the enzyme activity may be caused by tumor cells and soluble mediators secreted therefrom affecting functional activity of DCs and/or their monocyte precursors. In this case, high activity of TACE/ADAM-17 in DCs can also promote tumor growth via inhibition of the cytotoxic DC activity by way of decreasing mTNFα levels. However, inhibition of TACE/ADAM-17 reduces mTNFα shedding on IFN-DC membranes and enhances the cytotoxic DC activity against TNFα/TNF-R1-sensitive tumor cells [18].

Increased mTNFα levels [18] along with enhanced cytotoxic activity of IFN-DCs from glioblastoma patients against autologous tumor cells after treatment with rIL-2 and dsDNA position these mediators as activators of the mTNFα-mediated cytotoxic effect. Despite a similar downstream stimulatory effect, rIL-2 and dsDNA have different mechanisms of action on mTNFα expression by DCs. The stimulatory effect of dsDNA on DCs is associated with an increase from the baseline low TNFα mRNA levels, as well as with a reduction from the baseline high TACE/ADAM-17 enzyme levels and activity. On the contrary, the effect of rIL-2 is not associated with a significant increase in the TNFα mRNA level and concomitant suppression of TACE/ADAM-17 activity, which indicates the involvement of other mechanisms regulating mTNFα levels. IL-2 is known to have a pleiotropic effect on immune cells, which is mediated through a specific cellular receptor, IL-2R [38]. Previously, we demonstrated that IFN-DCs express a high affinity IL-2 receptor, CD25 [25]. These findings suggested that IFN-DCs are likely to be sensitive to modulating effect of IL-2. TNFα synthesis is known to be regulated by various protein kinases (protein kinase C, p38 MAP kinase), which ensure TNFα mRNA stability [39,40]. Notably, IL-2 is a positive regulator of these kinases in activated cells of the immune system [41,42,43]. Therefore, it can be suggested that the effect of rIL-2 on TNFα expression in IFN-DCs from glioblastoma patients is implemented at the post-transcriptional level via a protein kinase-dependent mechanism.

The mechanism of dsDNA action on DCs remains unclear. It was shown that upon entry into IFN-DC cytosol, the exogenous dsDNA is almost immediately transported to the nucleus [44]. Nuclear accumulation of dsDNA in IFN-DCs is likely to ensure direct activation of expression of various genes, including *TNFα*, while the effect on TACE/ADAM-17 could be indirect via targeting regulation of other molecules.

We demonstrated that administration of rIL-2 and dsDNA significantly increased the expression of perforin and granzyme B (dsDNA treatment), as well as tended to up-regulate FasL and TRAIL expression on patient-derived IFN-DCs. This data suggests that rIL-2 and dsDNA could be considered as activators of TNFα-independent cytotoxic activity of patient-derived IFN-DCs against autologous glioblastoma cells.

As far as study limitations are concerned, we did not use primary tumor cells but rather cell lines obtained from primary glioblastoma cultures as target cells to study sensitivity of glioblastoma to DC-mediated cytotoxic activity. Although specific properties of the tumor itself and inherent cellular relationships are retained in primary tumor cell cultures, cellular proliferation rate in such cultures is reduced, thus limiting the possibility of conducting some studies. Generation of cell lines from primary cultures allowed us to increase the number of tumor cells after several passages and obtain morphologically homogenous cell populations. In addition, glioblastoma is characterized by low mutational burden and low ability of neoantigen formation [45], and these properties can be preserved in cell lines derived from primary glioblastoma cell cultures [46].

Taken together, we conclude that the ability of DCs to lyse tumor cells is an important phenomenon. The data obtained characterized a direct cytotoxic effect of IFN-DCs on glioblastoma cells and demonstrated a selective (TNFα/TNF-R1-mediated) defect in this function in DCs derived from glioblastoma patients. We determined mechanism underlying reduction in DC-mediated cytotoxic potential in glioblastoma patients and identified targets of further studies focused on regulation of cytotoxic functions and the possibility of correcting cytotoxic properties of DCs in cancer patients. Generation of DCs that not only trigger immune responses, but also exhibit a high cytotoxic potential against tumor cells can potentially modify our clinical approaches to treatment and routes of administration of DC-based vaccines, as well as make it possible to achieve more positive clinical outcomes of immunotherapy. This study highlights the relevance of basic research in terms of improving our diagnostic and treatment potential (such as identification of suitable biomarkers, and attempts to formulate new pharmacological strategies [47]) towards patients harboring high-grade gliomas, who face a challenging surgical management and a never ending course of adjuvant therapies. The cytotoxic activity of DCs essentially facilitate tumor antigen capture and its subsequent presentation to T-cells and may be used as therapeutic tool in boosting of the immune response aimed at eliminating tumor cells.

## 4. Materials and Methods

### 4.1. Patients

The study included 43 patients with glioblastoma multiforme (25 men and 18 women aged 23 to 75 years, median 54 years; 27 patients with newly diagnosed glioma and 16 patients with recurrent glioma) treated at the Department of Neurosurgery of Novosibirsk, Institute of Traumatology and Orthopedics, in 2016–2019. The histological analysis of brain tumor tissues was performed according to the World Health Organization 2016 classification of tumors of the central nervous system. Ninety-seven age- and sex-matched healthy anonymous individuals were recruited as healthy controls. Informed consent was obtained from all patients and donors according to the Declaration of Helsinki. The study was approved by the local ethics committees of Institute of Fundamental and Clinical Immunology (protocol no. 28, dated 15 December 2015) and Institute of Traumatology and Orthopedics (protocol no. 1, dated 11 January 2016).

### 4.2. Generation of DCs

To generate DCs, plastic adherent mononuclear cells (predominantly monocytes ) isolated from peripheral blood by density gradient centrifugation using Ficoll-Paque (GE Healthcare, Freiburg, Germany) were cultured in RPMI-1640 medium (Sigma-Aldrich, Irvine, UK), supplemented with 0.3 mg/mL L-glutamine, 5 mM HEPES buffer, 100 µg/mL gentamicin and 2.5% fetal calf serum (FCS, Sigma-Aldrich) in the presence of rhGM-CSF (40 ng/mL, Sigma-Aldrich) and rhIFN-α (Roferon-A, 1000 U/mL, Roche, Switzerland) at 37 °C at 5% CO_2_ concentration for 4 days. DC maturation was induced by further exposure to a standard lipopolysaccharide concentration [48] (10 µg/mL, LPS, *E. coli* O114: B4, Sigma-Aldrich, Jerusalem, Israel) for additional 24 h. The viability of DCs determined by Trypan blue exclusion was more than 93–95% in all cases. The phenotypic characteristics of donor-derived and patient-derived IFN-DCs are shown in Table 1.

In some series of experiments, recombinant human interleukin-2 (rIL-2, 50 U/mL) and double-stranded human DNA (dsDNA) (5 μg/mL) were added concomitantly with LPS to cell cultures of patient DCs. Double-stranded DNA was obtained as described in previous reports [49]. Briefly, human DNA was isolated from placentas of healthy women using a phenol-free method. DNA was fragmented in an ultrasonic disintegrator at a frequency of 22 kHz to obtain a mixture of DNA fragments with a size of 200 to 6000 bp. DNA preparations were dissolved in saline and stored at −20 °C.

### 4.3. Tumor Cell Lines

Primary glioblastoma cell cultures were obtained by mechanical and enzymatic (0.3% collagenase, Sigma-Aldrich, Rehovot, Israel) disaggregation of tumor issues derived from patients with histologically verified glioblastoma multiforme (Grade IV, *n* = 13). Cell suspensions were cultured in DMEM/F12 medium (Gibco, Paisley, UK) supplemented with 0.3 mg/mL *L*-glutamine, 5 mM HEPES buffer, 10^−4^ M mercaptoethanol, 100 µg/mL gentamicin and 10% FBS. Cells were refreshed with new culture medium twice a week and maintained at 37 °C in an 5% CO_2_ incubator. Primary cell cultures were passaged upon reaching cellular subconfluence (70–80%) using 0.25% trypsin/EDTA (Sigma-Aldrich, St. Louis, MO, USA). Cells were further cultured as cell lines (*n* = 13) in complete culture medium. The passage number for cell lines derived from primary tumor cell cultures ranged from two to five.

Immortalized human glioblastoma U-87 cell line was obtained from Dr. Shilov (Federal Research Center Institute of Cytology and Genetics of Siberian Branch of the Russian Academy of Sciences) and cultured in αMEM medium (Gibco, Paisley, UK) supplemented with 100 µg/mL gentamicin and 10% FBS.

### 4.4. Flow Cytometry Analysis

Phenotype of DCs was analyzed by flow cytometry based on expression of surface molecules using fluorochrome-conjugated mAbs (BD Pharmingen™, San Jose, CA USA; R&D Systems, Minneapolis, MN, USA) specific for CD14 (FITC), CD83 (Pe), CD86 (FITC), HLA-DR (FITC), CCR7 (Pe), FasL (Pe), TRAIL (Pe), CD107a (APC) or TACE/ADAM-17 (APC). For intracellular molecule detection, IFN-DCs were permeabilized using Fixation/permeabilization solution kit (BD Cytofix/Cytoperm™, San Jose, CA, USA) according to the manufacturer′s instructions and stained with anti-CD107a- (APC, BD Pharmingen™, USA), anti-granzyme B-(Pe, BD Pharmingen™, USA), anti-perforin (Pe, BioLegend, San Diego, CA, USA) mAbs. In each experiment, isotype-matched control monoclonal antibodies were included to determine non-specific background staining. Cells were analyzed by flow cytometry using BD FACSCalibur flow cytometer (BD Biosciences, San Jose, CA, USA) and CellQuest analysis Program. A minimum of 10,000 events was collected from each cell sample.

Tumor cell phenotyping was performed by staining with anti-Fas (FITC) and anti-TRAIL-R2 (Pe) mAbs (BD Pharmingen™, USA) according to the standard procedure after the passage with 0.25% trypsin/EDTA. To assess proliferative activity of tumor cells, Pe-conjugated anti-Ki-67 mAbs (BD PharMingen, San Jose, CA, USA) and matched isotype control mAbs were used in combination with intracellular molecule staining.

### 4.5. MTT Assay

Glioblastoma cell lines derived from primary tumor cultures and U87 cell line (50 × 10^3^ cells/well) were cultured with LPS-stimulated donor IFN-DCs in 96-well flat-bottom plates at a ratio of 1:1 in triplicates. In some experiments, DCs were pre-incubated for 1 h with recombinant human TNFR1/TNFRSF1A Fc chimera protein (10 μg/mL, R & D Systems, USA), recombinant human rhFas/TNFRSF6/CD95 Fc chimera protein (10 μg/mL, R & D Systems, USA), recombinant human rhTRAIL R2/TNFRSF10B Fc chimera protein (10 μg/mL, R & D Systems, USA) or for 2 h with concanamicin A (100 nM, Sigma-Aldrich, Milano, Italy).

After 20 h incubation, MTT (3-(4,5-dimethylthiazol-2-yl)2,5-diphenyl tetrazolium bromide, Sigma-Aldrich, Saint Louis, MO, USA) solution (20 µL of 5 mg/mL stock) was added to each well, and the plates were incubated in the dark for 4 h at 37 °C. Then, the plates were centrifuged at 1500 rpm for 5 min, the medium was removed and dimethyl sulfoxide (150 µL, DMSO, MP Biomedicals, LLC, Illkirch Cedex, France) was added to each well to dissolve formazan crystals. Absorbance at 492 nm was measured using Multiwell spectrophotometer (Thermo Scientific Multiskan FC, Vantaa, Finland). The percentage of cytotoxicity (%) was calculated using the following formula: [1−(absorbance in experimental well with target and effector cells-background absorbance of effector cells/absorbance of untreated target cells)] × 100.

### 4.6. Apoptosis Assay

Glioblastoma cells stained with 5(6)-carboxyfluorescein diacetate N-succinimidyl ester (CFSE) fluorescent dye (2 μM; Molecular probes, Inc., Eugene, OR, USA) were incubated with IFN-DCs at a ratio DCs:tumor cells 10:1 for 18 h. Percentage of tumor cells apoptosis was measured using annexin V-APC apoptosis detection kit (BD Pharmingen™, USA). Briefly, cells were washed with PBS and labeled with APC-conjugated annexin V and propidium iodide (PI) for 15 min at room temperature, followed by analysis on a FACSCalibur flow cytometer. A minimum of 10,000 events within the CFSE-positive gate region were collected for each sample.

### 4.7. CD107a Degranulation Assay

IFN-DCs were incubated with glioblastoma cells pre-stained with CFSE dye at an DC:tumor cell ratio of 10:1 for 18 h in the presence of APC-conjugated anti-human CD107a and monensin A (10 μM) (all BD Pharmingen™). After co-culture with target glioblastoma cells, DCs were washed and analyzed on a FACS Calibur flow cytometer using CellQuest analysis Program. The frequency of degranulating DCs were measured as CD107a^+^ DCs gated within CFSE-negative cells. A minimum of 10,000 events within the gate region were collected for each sample.

### 4.8. TNFa mRNA Expression Assay

Total RNA preparation was isolated from IFN-DC samples of healthy donors and patients before and 2 h after LPS stimulation using a RIBO-sol-B commercial kit (FGNII CNIIE Rospotrebnadzor, Russia) according to the manufacturer’s instructions. RNA was quantified using a Nanodrop ND-100 spectrophotometer (NanoDrop Technologies, Inc., USA). Reverse transcription reaction was performed on the mRNA template using a C1000™ Thermal Cycler (Bio-Rad Laboratories, Inc., Hercules, CA, USA) and a MMLV RT kit (Eurogen, Moscow, Russia) according to the manufacturer’s protocol. Primers were selected with the Vector NTI program and synthesized by the Biosset company. Sequences of the primers used in real-time quantitative PCR (for, stands for the forward primer, rev, the reverse primer):

TNFα-for 5′-CCAATGGCGTGGAGCTGAGA-3′
TNFα-rev 5′-TGATGGTGTGGGTGAGGAGCAC-3′
RPLP0-for 5′-AGGCCTTCTTGGCTGATCCATCT-3′
RPLP0-rev 5′-TATCCTCGTCCGACTCCTCCGA-3′.

Real-time PCR was performed in 96-well plates using SYBR® Green PCR Master Mix reagents on a ViiATM device (Applied Biosystems, Foster City, CA, USA). Reaction conditions included initial denaturation (95 °C) and subsequent 40 replication cycles (95 °C 15 s, 58 °C 30 s and 72 °C 60 s). Each PCR product included triplets of the tested samples. The results of quantitative real-time PCR were analyzed using QuantStudioTM Real-Time PCR Software v1.1. mRNA level of *TNF*α gene was normalized to the large P0 subunit of the acidic ribosomal phosphorylated protein (RPLP0) with 2^−∆∆*C*t^ method. As a control group, IFN-DCs of healthy donors were used and the expression level of *TNF*α gene was taken as 1.

### 4.9. TNFα-Converting Enzyme (TACE/ADAM-17) Activity Assay

To assess TACE/ADAM-17 enzyme activity in DCs in response to stimulus (LPS, rIL-2, dsDNA), a commercial SensoLyte®520 TACE activity assay kit (Anaspec, Inc, Fremont, CA, USA) was used, according to the manufacturer’s instructions. Enzyme activity was measured judging by the intensity of fluorescence as detected by spectrofluorimetry. In parallel, protein concentration in the DC samples was determined by the Bradford method. The results were expressed in relative fluorescent units per 1 μg of protein in a given sample (RFU/μg protein).

### 4.10. Statistical Analysis

Statistical analysis was performed using Statistica 6.0 and GraphPad Prism 8.0 software. Data are presented as mean ± standard error of the mean (SE), as well as median (Me) and interquartile range (LQ–UQ, 25–75% quartile) for each group. Statistical differences were analyzed by Mann–Whitney *U* test or paired sign test. Correlations between variables were evaluated using Spearman rank correlation test. *p* value < 0.05 were considered statistically significant. Heatmap analysis was performed using GraphPad Prism 8.0 software.

## Figures and Tables

**Table 1:** The phenotypic characteristics of healthy donor and patient IFN-DCs.

DC Marker	Donor IFN-DCs	Glioblastoma IFN-DCs	*p*
CD14	15.0 ± 4.2	29.6 ± 8.0	0.032
CD83	26.2 ± 7.1	19.7 ± 4.7	0.041
HLA-DR	84.4 ± 4.6	83.5 ± 4.7	0.71
CD86	78.0 ± 4.7	75.5 ± 14.3	0.53
CCR7	12.7 ± 1.9	12.2 ± 1.1	0.66

LPS-stimulated IFN-DCs were analyzed by flow cytometry for expression of DC-related surface antigens. The relative percentage of positive cells (%) among the total DC population is given as mean ± SE. *p*-value indicates differences between the means.

**Table 2:** Cytotoxic activity of IFN-DCs against glioblastoma cells.

Cell Line	Donor DCs (Me (LQ-UQ) (%)	Autologous Patient DCs (%)	Impairment Index of Cytotoxicity of Patient IFN-DCs (%)	the Involvement of TNFα/TNF-R1-Signaling Pathway (Me, %)
GB#6	45.4 (41–55)	22.7	50.0	11
GB#7	69.8 (63.4–76.2)	46.6	33.2	40.1
GB#8	36.9 (23.6–49.5)	27.6	25.2	24.2
GB#9	46.8 (34.8–58.7)	37.5	19.9	17.5
GB#10	29.1 (23.0–43.5)	32.7	0	0
GB#11	47.2 (40.0–50.7)	27.8	41.1	27.7
GB#12	45.5 (37.3–49.4)	24.8	45.5	23.2
GB#13	60.8 (58.2–63.3)	75.9	0	0

Median (Me) and interquartile range (LQ-UQ) of cytotoxic activity (%) of donor IFN-DCs, and individual values of cytotoxic activity (%) of patient-derived IFN-DCs against glioblastoma cell lines (GB) are presented. Medians of cytotoxic activity impairment of patient-derived IFN-DC towards glioblastoma cell lines are calculated as: [1-(cytotoxicity of DC_patient_/Me cytotoxicity of DC_donor_)] × 100%. The involvement of TNFα/TNF-R1-signaling pathway (right column) represents a blocking effect of rTNF-R1 on donor DC cytotoxicity, calculated as: [1-(cytotoxicity of DC_+rTNF-R1_/cytotoxicity of DC_control_)] × 100%.

## References

[1] Ni K., O’Neill H. (1997). The role of dendritic cells in T cell activation. Immunol. Cell Biol..

[2] Austyn J.M. (1998). Dendritic cells. Curr. Opin. Hematol..

[3] Fanger N.A., Maliszewski C.R., Schooley K., Griffith T.S. (1999). Human dendritic cells mediate cellular apoptosis via tumor necrosis factor-related apoptosis-inducing ligand (TRAIL). J. Exp. Med..

[4] Hira S.K., Mondal I., Bhattacharya D., Gupta K.K., Manna P.P. (2015). Downregulation of STAT3 phosphorylation enhances tumoricidal effect of IL-15-activated dendritic cell against doxorubicin-resistant lymphoma and leukemia via TNF-α. Int. J. Biochem. Cell Biol..

[5] Korthals M., Safaian N., Kronenwett R., Maihöfer D., Schott M., Papewalis C., Diaz Blanco E., Winter M., Czibere A., Haas R. (2007). Monocyte derived dendritic cells generated by IFN-alpha acquire mature dendritic and natural killer cell properties as shown by gene expression analysis. J. Transl. Med..

[6] Stary G., Bangert C., Tauber M., Strohal R., Kopp T., Stingl G. (2007). Tumoricidal activity of TLR7/8-activated inflammatory dendritic cells. J. Exp. Med..

[7] Van den Bergh J.M.J., Guerti K., Willemen Y., Lion E., Cools N., Goossens H., Vorsters A., Van Tendeloo V.F.I., Anguille S., Van Damme P. (2014). HPV vaccine stimulates cytotoxic activity of killer dendritic cells and natural killer cells against HPV-positive tumour cells. J. Cell. Mol. Med..

[8] Lu G., Janjic B.M., Janjic J., Whiteside T.L., Storkus W.J., Vujanovic N.L. (2002). Innate direct anticancer effector function of human immature dendritic cells. II. Role of TNF, Lymphotoxin- 1 2, Fas Ligand, and TNF-Related Apoptosis-Inducing Ligand. J. Immunol..

[9] Koya T., Yanagisawa R., Higuchi Y., Sano K., Shimodaira S. (2017). Interferon-α-inducible dendritic cells matured with OK-432 exhibit TRAIL and Fas Ligand pathway-mediated killer activity. Sci. Rep..

[10] Sallusto F., Lanzavecchia A. (1994). Efficient presentation of soluble antigen by cultured human dendritic cells is maintained by granulocyte/macrophage colony-stimulating factor plus interleukin 4 and downregulated by tumor necrosis factor alpha. J. Exp. Med..

[11] González-Navajas J.M., Lee J., David M., Raz E. (2012). Immunomodulatory functions of type I interferons. Nat. Rev. Immunol..

[12] Gessani S., Conti L., Del Cornò M., Belardelli F. (2014). Type I interferons as regulators of human antigen presenting cell functions. Toxins.

[13] Paquette R.L., Hsu N.C., Kiertscher S.M., Park A.N., Tran L., Roth M.D., Glaspy J.A. (1998). Interferon-alpha and granulocyte-macrophage colony-stimulating factor differentiate peripheral blood monocytes into potent antigen-presenting cells. J. Leukoc. Biol..

[14] Santini S.M., Lapenta C., Logozzi M., Parlato S., Spada M., Di Pucchio T., Belardelli F. (2000). Type I interferon as a powerful adjuvant for monocyte-derived dendritic cell development and activity in vitro and in Hu-Pbl-Scid mice. J. Exp. Med..

[15] Lapenta C., Santini S.M., Spada M., Donati S., Urbani F., Accapezzato D., Franceschini D., Andreotti M., Barnaba V., Belardelli F. (2006). IFN-α-conditioned dendritic cells are highly efficient in inducing cross-priming CD8+ T cells against exogenous viral antigens. Eur. J. Immunol..

[16] Jin Z., Fan J., Zhang Y., Yi Y., Wang L., Yin D., Deng T., Ye W. (2017). Comparison of morphology, phenotypes and function between cultured human IL-4-DC and IFN-DC. Mol. Med. Rep..

[17] Tyrinova T.V., Leplina O.Y., Mishinov S.V., Tikhonova M.A., Shevela E.Y., Stupak V.V., Pendyurin I.V., Shilov A.G., Alyamkina E.A., Rubtsova N.V. (2013). Cytotoxic activity of ex-vivo generated IFNα-induced monocyte-derived dendritic cells in brain glioma patients. Cell. Immunol..

[18] Tyrinova T., Leplina O., Mishinov S., Tikhonova M., Kalinovskiy A., Chernov S., Dolgova E., Stupak V., Voronina E., Bogachev S. (2018). Defective Dendritic Cell Cytotoxic Activity of High-Grade Glioma Patients’ Results from the Low Expression of Membrane TNFα and Can Be Corrected In Vitro by Treatment with Recombinant IL-2 or Exogenic Double-Stranded DNA. J. Interf. Cytokine Res..

[19] Hanif F., Muzaffar K., Perveen K., Malhi S.M., Simjee S.U. (2017). Glioblastoma multiforme: A review of its epidemiology and pathogenesis through clinical presentation and treatment. Asian Pac. J. Cancer Prev..

[20] Tyrinova T.V., Mishinov S.V., Leplina O.Y., Alshevskaya A.A., Kurochkina Y.D., Oleynik E.A., Kalinovskiy A.V., Lopatnikova Y.A., Chernov S.V., Stupak V.V. (2018). Role of TNFα/TNF-R1-signaling pathway in cytotoxic activity of dendritic cells against glioblastoma cell lines. Med. Immunol..

[21] Aggarwal B.B. (2003). Signalling pathways of the TNF superfamily: A double-edged sword. Nat. Rev. Immunol..

[22] Knight M.J., Riffkin C.D., Muscat A.M., Ashley D.M., Hawkins C.J. (2001). Analysis of FasL and TRAIL induced apoptosis pathways in glioma cells. Oncogene.

[23] Kataoka T., Takaku K., Magae J., Shinohara N., Takayama H., Kondo S., Nagai K. (1994). Acidification is essential for maintaining the structure and function of lytic granules of CTL. Effect of concanamycin A, an inhibitor of vacuolar type H(+)-ATPase, on CTL-mediated cytotoxicity. J. Immunol..

[24] Kataoka T., Shinohara N., Takayama H., Takaku K., Kondo S., Yonehara S., Nagai K. (1996). Concanamycin A, a powerful tool for characterization and estimation of contribution of perforin- and Fas-based lytic pathways in cell-mediated cytotoxicity. J. Immunol..

[25] Leplina O.Y., Tyrinova T.V., Tikhonova M.A., Ostanin A.A., Chernykh E.R. (2015). Interferon alpha induces generation of semi-mature dendritic cells with high pro-inflammatory and cytotoxic potential. Cytokine.

[26] Black R.A., Rauch C.T., Kozlosky C.J., Peschon J.J., Slack J.L., Wolfson M.F., Castner B.J., Stocking K.L., Reddy P., Srinivasan S. (1997). A metalloproteinase disintegrin that releases tumour-necrosis factor-α from cells. Nature.

[27] Friese M.A., Platten M., Lutz S.Z., Naumann U., Aulwurm S., Bischof F., Bühring H.-J., Dichgans J., Rammensee H.-G., Steinle A. (2003). MICA/NKG2D-mediated immunogene therapy of experimental gliomas. Cancer Res..

[28] Wischhusen J., Friese M.A., Mittelbronn M., Meyermann R., Weller M. (2005). HLA-E protects glioma cells from NKG2D-mediated immune responses in vitro: Implications for immune escape in vivo. J. Neuropathol. Exp. Neurol..

[29] Wu A., Wiesner S., Xiao J., Ericson K., Chen W., Hall W.A., Low W.C., Ohlfest J.R. (2007). Expression of MHC I and NK ligands on human CD133+ glioma cells: Possible targets of immunotherapy. J. Neurooncol..

[30] Kato T., Sawamura Y., Tada M., Sakuma S., Sudo M., Abe H. (1995). p55 and p 75 tumor necrosis factor receptor expression on human glioblastoma cells. Neurol. Med. Chir. (Tokyo).

[31] Saggioro F.P., Neder L., Stávale J.N., Paixão-Becker A.N.P., Malheiros S.M.F., Soares F.A., Pittella J.E.H., Matias C.C.M.S., Colli B.O., Carlotti C.G. (2014). Fas, FasL, and cleaved caspases 8 and 3 in glioblastomas: A tissue microarray-based study. Pathol. Res. Pract..

[32] Wang S.-S., Feng L., Hu B.-G., Lu Y.-F., Wang W.-M., Guo W., Suen C.-W., Jiao B.-H., Pang J.-X., Fu W.-M. (2017). miR-133a promotes TRAIL resistance in glioblastoma via suppressing death receptor 5 and activating NF-κB signaling. Mol. Ther. Nucleic Acids.

[33] Gratas C., Tohma Y., Van Meir E.G., Klein M., Tenan M., Ishii N., Tachibana O., Kleihues P., Ohgaki H. (1997). Fas Ligand expression in glioblastoma cell lines and primary astrocytic brain tumors. Brain Pathol..

[34] Shinohara H., Yagita H., Ikawa Y., Oyaizu N. (2000). Fas drives cell cycle progression in glioma cells via extracellular signal-regulated kinase activation. Cancer Res..

[35] Bellail A.C., Tse M.C.L., Song J.H., Phuphanich S., Olson J.J., Sun S.Y., Hao C. (2010). DR5-mediated DISC controls caspase-8 cleavage and initiation of apoptosis in human glioblastomas. J. Cell. Mol. Med..

[36] Capper D., Gaiser T., Hartmann C., Habel A., Mueller W., Herold-Mende C., von Deimling A., Siegelin M.D. (2009). Stem-cell-like glioma cells are resistant to TRAIL/Apo2L and exhibit down-regulation of caspase-8 by promoter methylation. Acta Neuropathol..

[37] Wu B., Sha L., Wang Y., Xu W., Yu Y., Feng F., Sun C., Xia L. (2014). Diagnostic and prognostic value of a disintegrin and metalloproteinase-17 in patients with gliomas. Oncol. Lett..

[38] Boyman O., Sprent J. (2012). The role of interleukin-2 during homeostasis and activation of the immune system. Nat. Rev. Immunol..

[39] Carpenter S., Ricci E.P., Mercier B.C., Moore M.J., Fitzgerald K.A. (2014). Post-transcriptional regulation of gene expression in innate immunity. Nat. Rev. Immunol..

[40] Chung Y.-J., Zhou H.-R., Pestka J.J. (2003). Transcriptional and posttranscriptional roles for p38 mitogen-activated protein kinase in upregulation of TNF-alpha expression by deoxynivalenol (vomitoxin). Toxicol. Appl. Pharmacol..

[41] Crawley J.B., Rawlinson L., Lali F.V., Page T.H., Saklatvala J., Foxwell B.M.J. (1997). T cell proliferation in response to interleukins 2 and 7 requires p38MAP kinase activation. J. Biol. Chem..

[42] Fairhurst R.M., Daeipour M., Amaral M.C., Nel A.E. (1993). Activation of mitogen-activated protein kinase/ERK-2 in phytohaemagglutin in blasts by recombinant interleukin-2: Contrasting features with CD3 activation. Immunology.

[43] Gollob J.A., Schnipper C.P., Murphy E.A., Ritz J., Frank D.A. (1999). The functional synergy between IL-12 and IL-2 involves p38 mitogen-activated protein kinase and is associated with the augmentation of STAT serine phosphorylation. J. Immunol..

[44] Alyamkina E.A., Dolgova E.V., Likhacheva A.S., Rogachev V.A., Sebeleva T.E., Nikolin V.P., Popova N.A., Kiseleva E.V., Orishchenko K.E., Sakhno L.V. (2010). Exogenous allogenic fragmented double-stranded DNA is internalized into human dendritic cells and enhances their allostimulatory activity. Cell. Immunol..

[45] Garg A.D., Vandenberk L., Van Woensel M., Belmans J., Schaaf M., Boon L., De Vleeschouwer S., Agostinis P. (2017). Preclinical efficacy of immune-checkpoint monotherapy does not recapitulate corresponding biomarkers-based clinical predictions in glioblastoma. Oncoimmunology.

[46] Howley R., Kinsella P., Buckley P.G., Alcock L., Jansen M., Heffernan J., Stallings R.L., Brett F.M., Amberger-Murphy V., Farrell M.A. (2012). Comparative genomic and proteomic analysis of high grade glioma primary cultures and matched tumor in situ. Exp. Cell Res..

[47] Ganau M., Paris M., Syrmos N., Ganau L., Ligarotti G., Moghaddamjou A., Prisco L., Ambu R., Chibbaro S. (2018). How Nanotechnology and Biomedical Engineering Are Supporting the Identification of Predictive Biomarkers in Neuro-Oncology. Medicines.

[48] Loison E., Gougeon M.-L. (2014). Thimerosal compromises human dendritic cell maturation, IL-12 production, chemokine release, and T-helper polarization. Hum. Vaccin. Immunother..

[49] Alyamkina E.A., Nikolin V.P., Popova N.A., Minkevich A.M., Kozel A.V., Dolgova E.V., Efremov Y.R., Bayborodin S.I., Andrushkevich O.M., Taranov O.S. (2015). Combination of cyclophosphamide and double-stranded DNA demonstrates synergistic toxicity against established xenografts. Cancer Cell Int..

